# Multi-wavelength interference phase imaging for automatic breast cancer detection and delineation using diffuse reflection imaging

**DOI:** 10.1038/s41598-023-50475-9

**Published:** 2024-01-03

**Authors:** Alaaeldin Mahmoud, Yasser H. El-Sharkawy

**Affiliations:** Optoelectronics and Automatic Control Systems Department, Military Technical College, Kobry El-Kobba, Cairo, Egypt

**Keywords:** Cancer, Oncology, Optics and photonics

## Abstract

Millions of women globally are impacted by the major health problem of breast cancer (BC). Early detection of BC is critical for successful treatment and improved survival rates. In this study, we provide a progressive approach for BC detection using multi-wavelength interference (MWI) phase imaging based on diffuse reflection hyperspectral (HS) imaging. The proposed findings are based on the measurement of the interference pattern between the blue (446.6 nm) and red (632 nm) wavelengths. We consider implementing a comprehensive image processing and categorization method based on the use of Fast Fourier (FF) transform analysis pertaining to a change in the refractive index between tumor and normal tissue. We observed that cancer growth affects tissue organization dramatically, as seen by persistently increased refractive index variance in tumors compared normal areas. Both malignant and normal tissue had different depth data collected from it that was analyzed. To enhance the categorization of ex-vivo BC tissue, we developed and validated a training classifier algorithm specifically designed for categorizing HS cube data. Following the application of signal normalization with the FF transform algorithm, our methodology achieved a high level of performance with a specificity (*Spec*) of 94% and a sensitivity (*Sen*) of 90.9% for the 632 nm acquired image categorization, based on preliminary findings from breast specimens under investigation. Notably, we successfully leveraged unstained tissue samples to create 3D phase-resolved images that effectively highlight the distinctions in diffuse reflectance features between cancerous and healthy tissue. Preliminary data revealed that our imaging method might be able to assist specialists in safely excising malignant areas and assessing the tumor bed following resection automatically at different depths. This preliminary investigation might result in an effective "in-vivo" disease description utilizing optical technology using a typical RGB camera with wavelength-specific operation with our quantitative phase MWI imaging methodology.

## Introduction

BC is the most prevalent type of cancer in the world and the main reason why women die from cancer^1^. This disease was contributing for almost 570 thousand deaths in the last five years^2^. Furthermore, with 2.3 million newly diagnosed cases on average have been with malignant tumors annually^3,4^. Invasive BC tumor cell that can expand to various parts of the body such as the brain, liver, bone, and lungs, indicating its severity^5^. Consequently, one of the critical clinical challenges in BC management is the need for precise intraoperative margin assessment during BC surgery. This challenge arises from the complex nature of breast tissue, where ensuring cancer-free margins is essential to minimize the risk of recurrence and improve patient outcomes. Due to the lack of adequate operational tumor edge assessment techniques, complete tumor evacuation is tough^6^. As a result, a tumor is discovered at the resected edge of the excised specimen, and upwards to 37% of female patients undergoing breast-conserving surgeries are affected by this problem^7^. This considerable amount of tumor is still present in the patient, increasing the threat of a tumor recurrence and decreasing the likelihood of long-term disease-specific survival^8^. For efficient treatment and better results, early detection and correct diagnosis, it is crucial to develop new quantitative approaches that combine imaging and processing that can provide a more accurate diagnosis of BC. Many techniques have been developed to achieve this based on the light interaction with different specimens. Although phase contrast microscopy^9^ alleviated the issue of delivering in-focus phase contrast image, phase cannot be distinguished from intensity in the generated imaging; hence the method is not quantifiable. In order to resolve such constraints, significant work has been committed in past few years to creating quantitative imaging based on phases approaches, in which light path information all over a specimen is quantified. It has been demonstrated that the specifics of the relationship between light and tissue may be fully understood by considering the phase and amplitude of an optical field transmitted through tissues, including scattering properties. Many traditional coherent interference techniques like holography are used to quantify phase. However, interferometric devices are frequently massive, necessitate a complex optical system, and struggle from noise and diffraction limitations^10^.There is also a non-interferometric setup solution, quantitative phase imaging^11–17^, which has less stringent experimental requirements than interferometric methodologies. Using polychromatic radiation, highly structured samples can be precisely phase characterized. However, this method necessitates sample displacement through the focus and extensive computations, which may limit its applicability to interactive biomedical research. This solution has also the drawback of requiring a lot of measurements. These limitations might be overcome by applying HS imaging, which doesn’t require making contact with the tissue or the use of exogenous contrast chemicals. HS imaging can quickly quantify the whole resection edge at specific wavelengths^18^. This technique enables non-invasive identification of BC by analyzing the spectral properties of the tissue. Compared to traditional diagnostic methods, HS imaging can provide a more comprehensive view of the tissue. By examining the spectral data, we could obtain information on characteristics of the tissue, which can be used to distinguish between healthy and malignant areas. Moreover, this approach is highly sensitive and specific, allowing for early detection of cancerous growths and an accurate diagnosis of the disease. There are many researchers who have applied HS imaging for breast malignant tissue early diagnosis. Researchers in^19^ explore the evolution of medical imaging from traditional anatomical visualization to advanced molecular imaging techniques. They confirmed the importance of the use of diffuse reflectance spectroscopy (DRS) in the context of intraoperative BC margin assessment using HS imaging. In Ref.^20^, the study aims to use a HS camera to capture the spectral signatures of malignant and normal breast tissue in the 400–1000 nm range for therapeutic and diagnostic purposes. The system measures the tissue's diffuse reflectance and light transmission through two exploratory modes. In Ref.^21^, K-means clustering approach was employed to group the BC location based on the amplitude computations. Another study employed HS imaging data from stimulated BC specimens to identify malignancies by observing changes in fluorescence characteristics when compared to normal tissue^22^. The research study in Ref.^23^ investigates the use of DRS to distinguish malignant breast tissue from healthy tissue, comparing an extended wavelength range (450–1550 nm) to the standard range (450–900 nm). The results indicate that extended-wavelength DRS, with a *Sen* of 94% and a *Spec* of 91%, reflects the superiority of applying diffuse reflectance modalities. Multi-wavelength interference, a prospective BC detection technique, makes advantage of the interference pattern produced by dividing a light beam into a reference beam and a sample beam^24,25^. By detecting the phase distinction between the two beams, BC and other tissue properties may be identified^26,27^. Our study demonstrates that the phase difference between the specimen and reference beams is determined by analyzing the interference patterns between different light wavelengths in MWI using the captured cube image by HS imaging. With the use of this phase information, 3D maps of the tissue's thickness and refractive index may be created, which can reveal the existence of tumors. For instance, since tumors have higher refractive indices than healthy tissue^28^, increases in the refractive index of breast tissue may be a sign of breast cancer. Furthermore, the utilization of various wavelengths can provide details about the tissue's absorption and scattering characteristics, which can be utilized to differentiate between various tissue kinds and structures with high-sensitivity outcomes.

In this study, we provide an automated method for BC detection using MWI phase imaging based on diffuse reflection HS imaging. Meanwhile, after being exposed to polychromatic uniform light, we measured the depth of variation of dispersed light intensity and the associated phase shift at fluctuation frequency. The difference in refractive indices between two media affects how quickly light travels through them. The extracted phase shift change is reliant on the optical path differential variance between normal and malignant tissue. Due to the fact that refractive indices tend to drop as light wavelengths rise, more penetration of longer wavelength light (λ) than smallest^29,30^. We were able to find the tumor at various levels of the specimen using our MWI imaging techniques and the 3-D image the HS camera captured. Our findings show that the use of blue light can help to reduce scattering in the tissue, which can improve image quality, while the use of red light can provide deeper penetration into the tissue. By combining the two wavelengths, a more comprehensive view of the tissue can be obtained.

Our primary contribution lies in the utilization of HS camera, a cutting-edge technology that offers a wealth of information for biomedical applications. HS imaging provides us with a cube image containing 128 frames, each captured at different reflected wavelengths. These distinct wavelengths correspond to varying velocities of light as it interacts with examined breast tissue. As light travels through tissue, it creates interference patterns relative to a white paper reference, which serves as a critical benchmark for our measurements. The key to our approach is recognizing that the refractive index of tissue varies between tumor and normal breast tissue. Leveraging this variance, we employ an advanced algorithm based on FF transform to extract the phase information embedded within the interference patterns. This phase information becomes instrumental in our ability to differentiate between tumor and normal breast tissue. Moreover, we could harness the power of utilizing the cube image obtained from our HS camera. We employ this image to construct MWI patterns relative to the white paper reference, which acts as a stable and well-characterized surface. Subsequently, we deploy the FF transform algorithm to meticulously extract the phase information, thus enabling the differentiation of tumor and normal breast tissue. This innovative approach empowers us to aiding in the identification and delineation of BC. The significance of the white paper reference in this context cannot be overstated. It serves as a critical point of reference for our phase measurements, ensuring consistency and accuracy. By referencing all our measurements to this standardized surface, we effectively eliminate systematic errors and provide a basis for quantitative phase analysis. The use of a white paper reference allows us to quantitatively analyze the phase information relative to a known and consistent surface. This is essential for our research, as it enables us to differentiate between the phase shifts caused by the tissue samples and those caused by the reference surface. These phase differences provide valuable insights into the properties of the examined breast tissue. Furthermore, the white paper reference allows for reproducibility, comparison across different specimens, and the removal of confounding factors, thus playing a pivotal role in our methodology's success.

The proposed method relies on the assessment of the interference pattern between the blue and red wavelengths and the use of 2-D FF transform analysis to extract the phase information. The advantages of this technique include its non-invasive nature, high sensitivity, and ability to provide real-time imaging which could be classified between the tumor and the surrounding normal tissue. Our MWI imaging technique, combined with our FF transform approach, supports current efforts to achieve more objective tumor resection margin accuracy. With our method, pathologist might result in improvements to automatic optical BC diagnosis.

## Light interaction with tissues

It is necessary to utilize intensity measurements to compute the phase differences of the light. Phase variations are essential for surface profiling, particularly in biomedical imaging because they provide important details about an object's structure. A portion of the light that strikes a tissue's surface is absorbed by the tissue, while the remaining portion is scattered several times. Bulk scattering is a crucial factor in the dispersion of a significant portion of radiation in the backward direction, whereas multiple scattering and absorbance are accountable for the widening and eventual decay of a light beam as it passes through a tissue^31^.This will finally manifest as a diffuse reflectance signal that emanates from the tissue surface and conveys data on the structure and biochemistry of the tissue^32,33^.The refractive indices of the various tissue elements are not uniform, which causes light to be deflected. When light passes from one medium to another, its velocity changes proportionally to the refractive index variances between the two^34,35^. The median refractive index *n*_*m*_ of a tissue is determined by the volume concentration of the scatterers (*c*_*sc*_), the refractive indices of the ground matter (*n*_*g*_) and its scattering centers (*n*_*sc*_)^36^,

1 $${n}_{m}={c}_{sc}{n}_{sc}+\left(1-{c}_{sc}\right){n}_{g}.$$

**Figure 1:**
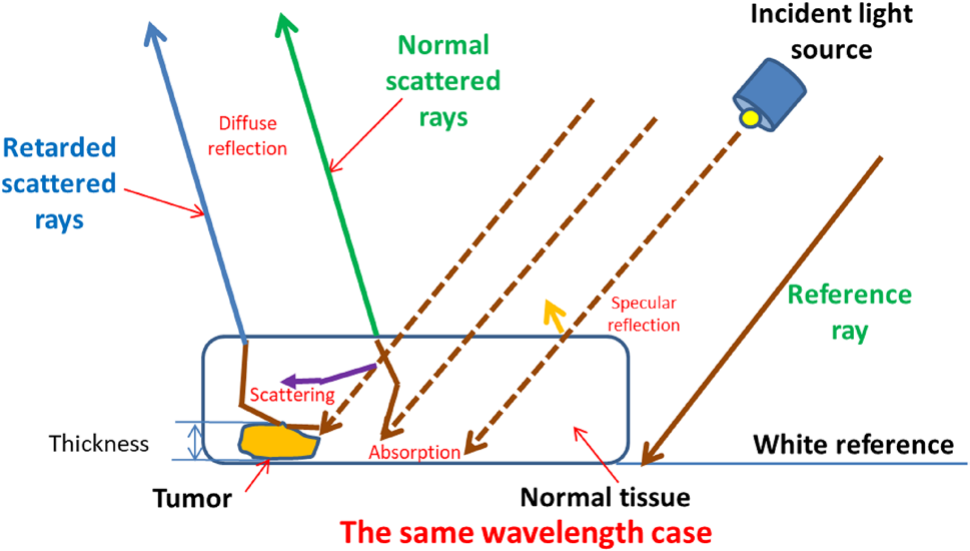
Phase wavefront change due to the change in optical path difference between normal tissue and tumor.

**Figure 2:**
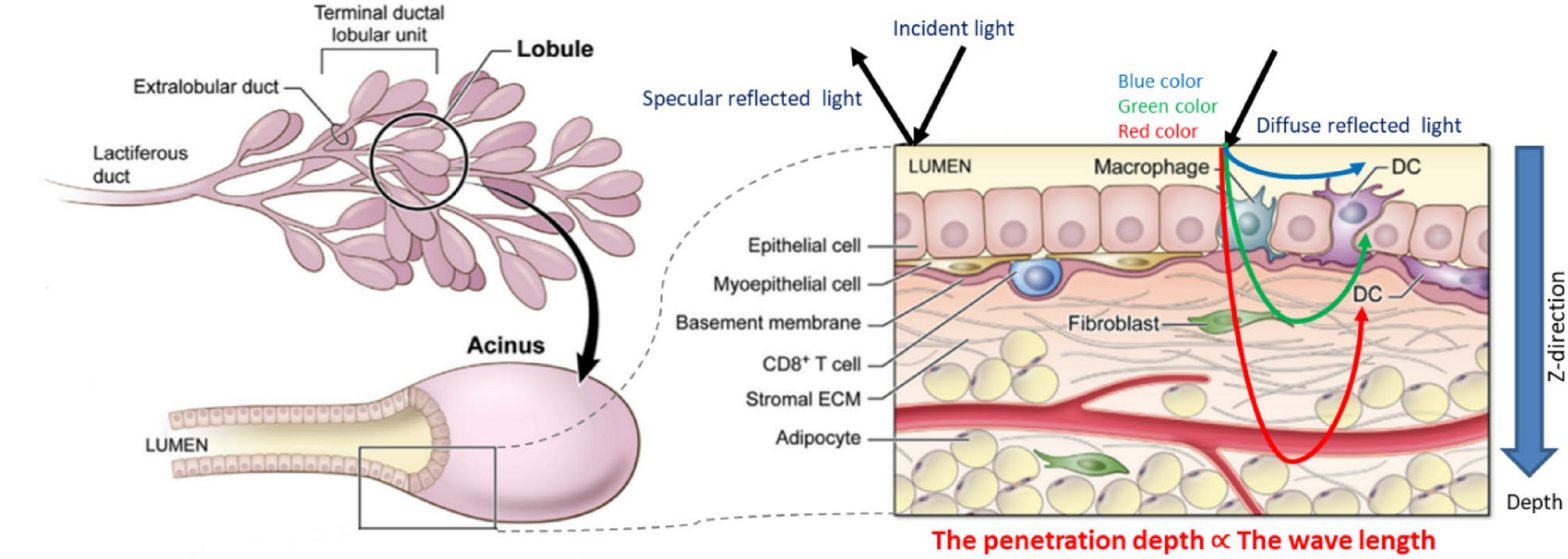
Longer wavelength light penetrated deeper into tissue than shorter wavelength light.

The scattering coefficients (µ_sc_) in a standard mono-disperse modeling of scattered dielectric spheres could be determined based on their relationship to the refractive index as^37^,

2 $${\mu }_{sc}=3.28\pi {r}^{2}{V}_{S}{[2\pi r/\lambda ]}^{0.37}{\left(\frac{{n}_{sc}}{{n}_{g}}-1\right)}^{2.09},$$

where V_S_ is the sphere volume density, λ is the wavelength and, r is the radius of sphere. Each of the tumor, normal, and reference diffuse reflected spectra had magnitude and phase angle variations, as illustrated in Fig. 1 and in accordance with the EM wave theory^38^. Both constructive and destructive waveform interferences are formed depending on how the wave interacts with the specimen. A positive interference happens if the waves are in phase. The interference is entirely destructive when two interfering waves be phase-shifted to maximize when one wave has reached its lowest^39^. Figure 2 shows light penetration into breast tissue demonstrating the difference in depth penetration with regard to wavelengths.

As depicted in Fig. 1, a refractive index difference exists between malignant and normal breast tissue. This variance leads to the presence of delayed scattered rays, which are light rays that have experienced a delay or phase shift due to their interaction with the tissue samples. These rays undergo a phase change as they traverse the tissue, and we leverage this phase information in our MWI technique to distinguish between tumor and normal breast tissue. Figure 2 illustrates how the difference in depth penetration between wavelengths directly contributes to the 3D phase shift data that we employ in our analysis.

## Materials and methods

Between October 2019 and February 2020, the investigation took place at the "Kobri El Koba Military Complex Hospital." The work received ethical approval from the Faculty of Medicine at Ain Shams University in Egypt and complied with the Declaration of Helsinki's Ethical Principles for Medical Research Involving Human Subjects. P.T.REC/009/003156 is the reference number. Before data collection started, each respondent read and agreed to two copies of written agreement. Ten females who had breast cancer growth underwent a breast-conserving surgery. Following the rigorous procedure, the patients were randomly selected, and breast tumor samples were obtained for neurological evaluation. The tumors were prepared for HS imager from the removed breasts after breast concealment. Breast tumor HS images were collected. These experimental breast tissue samples were sliced and placed in an ice box with deionized saline with measurements of (200 mm × 300 mm) and sample thickness of 3 ~ 5 mm. This extracted biopsy consists of normal tissue and the tumor. Analysis was conducted at 25 °C, a standard sample temperature of 23 to 25 degrees Celsius predicted before each preparatory and kept in the fridge up to − 70 °C. It was generally accepted that the region 50–100 mm away from the tumor was healthy, and pathology results supported this belief.

**Figure 3:**
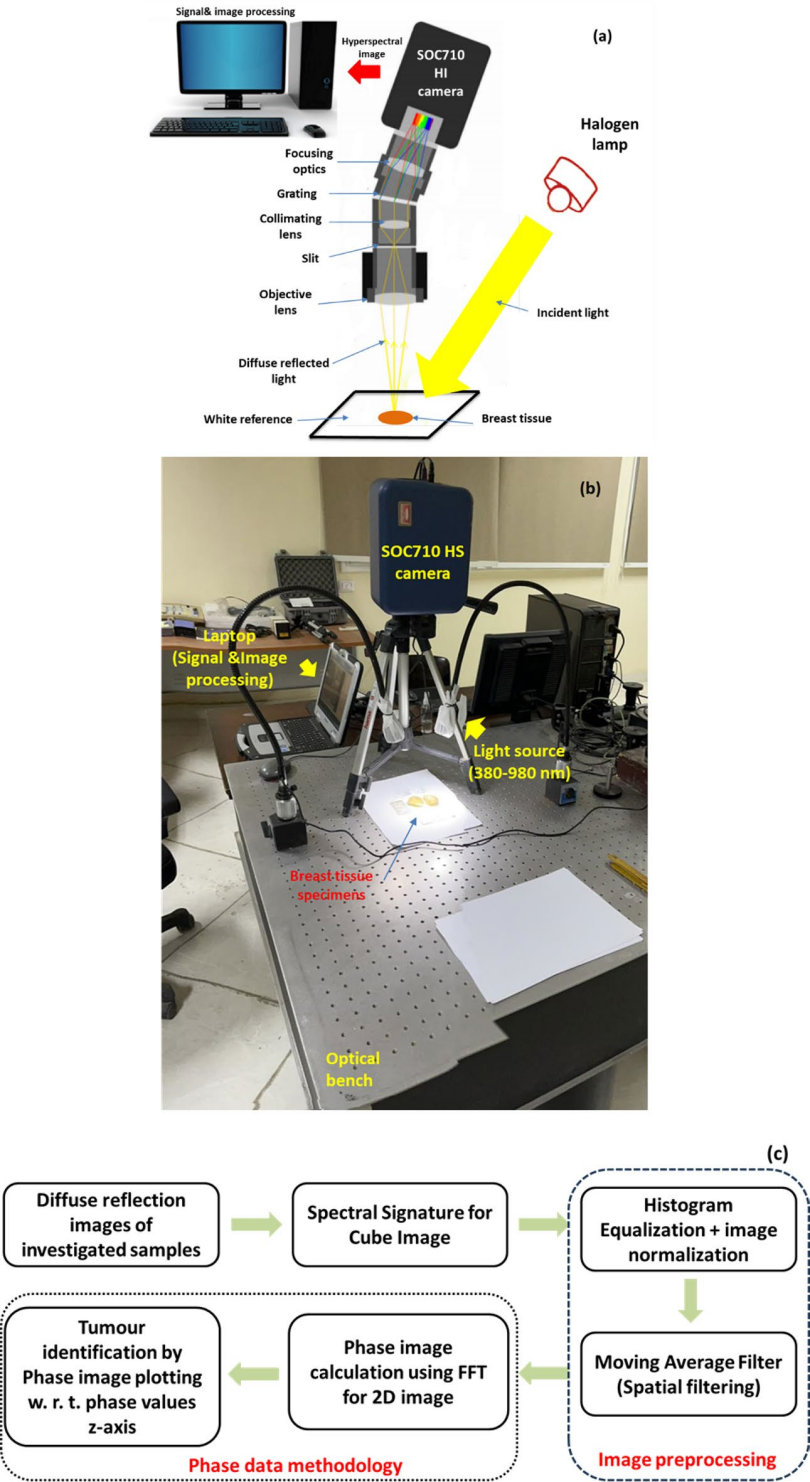
(**a**) Prototype of an HS optical image acquisition; (**b**) The HS benchtop arrangement used for the BC characterization; (**c**) Diagram of procedures for image processing and tool modules for this investigation utilizing the HS system.

The suggested imaging mechanism consists of a source light (Derungs, 20 P SX, Germany) having a wavelength region of 380–980 nm with line scanning and a HS camera (Surface Optics, SOC710, USA) with a VIS/NIR spectral region (380–1050 nm)^40^. The camera's installed lens is (Schneider, 400–1000 nm, Germany). Figure 3a provides a schematic illustration of the HS configuration. With a spatial resolution of under 40 microns and a spectral resolution of 5 nm, each collected cube picture included 128 spectral frames. As a result, the system was lit, and every component was fixed for the duration of the study times. The utilized optical lens had a field of view (FOV) of 10°, capturing a picture with dimensions of 6 cm × 8 cm at 50 cm, which is appropriate for a high focusing for the HS camera and the analyzed samples. Figure 3b illustrates the lab setup for our BC diagnosis to detect the malignant regions using the proposed phase analysis approach with HS images. The chart image recognition route that produced our promising benefits, analysis, and final finding is shown in Fig. 3c. A device (laptop) that runs software (HS-Analysis TM Data Analysis) managed the linear scanner's motors, adjusted exposure, and gathered the diffuse reflection characteristics data.

Using our FF transform approach, we completely converted the normalized spectral image to the phase domain, as shown in our suggested imaging approach in Fig. 3c. Our method is quicker and maintains all the raw data since it does waveform interpretation with less computation. Before converting the output spectrum images to the frequency domain, our preprocessing approach plays a pivotal role in ensuring the accuracy and reliability of the subsequent data analysis. The specific steps within this preprocessing sequence, which includes histogram equalization, image normalization, and the application of a moving average (MA) filtering, are strategically designed to enhance the quality of the data and prepare it for further analysis. Histogram equalization^41,42^ method's fundamental principle is to disperse the image's intensity values over a wider range to make the features in darker areas more obvious. Image normalization is a crucial component of our preprocessing approach, as it serves to standardize the data. The complex raw data from the cube image is transformed into a consistent 256-bit format^42^. This normalization process ensures that the data is ready for further analysis and that any variations in intensity are removed, allowing for more robust quantitative phase measurements. In order to further denoise and reconstruct the image, the following formula was applied to our MA filtering in the following phase^41,43^,

3 $$F\left(x,y\right)= \frac{1}{n \times m}+ {\sum }_{(k,z)\in w}^{\infty }NI(k,z),$$

where the operation is performed via a size of ‘*n* x *m*’, the recovered image is denoted by *F(x,y)*, *NI* is the noisy image ", and 'z' and 'k' stands for the window's ‘w’ column and row coordinates, respectively. This spatial filtering step is designed to enhance the quality of the cube image by reducing spatial noise and improving the overall data quality, which is crucial for our phase data clustering approach. The spectral aspects of our data are processed separately to extract the necessary phase information, and the MA filtering does not interfere with this spectral analysis. We may then use our Inv FF transform approach to get our original processed pictures in the time domain. After this, we obtained image information for phase using characteristics calculations. For the purpose of detecting tumors, phase shift outcomes are computed effectively. We could make a plotting for the studied breast specimens and track the BC for successive layers related to different depths according to the diffuse reflected spectra of both tumor and normal tissue using MWI approach with HS imager setup. The image processing algorithm sequencing was mostly based on the DADiSP 6.5 (DSP Development Corporation, USA) tool. By contrasting the results with the findings of the histology examinations, the effectiveness of the system analysis techniques offered is demonstrated. Equations (4), (5), and (6) illustrate three numerical values that may be assessed to examine the proposed MWI phase analysis technique based on the outcomes of these comparisons: *Sen*, *Spec*, and accuracy^21,41^.

4 $$Sen= \frac{TP}{TP+FN},$$

5 $$Spec = \frac{TN}{TN+FP},$$

6 $$accuracy= \frac{TP+TN}{TP+TN+FP+FN},$$

whereas False Negative (FN) occurrences are those in which the framework uncovers previously undetected masses, False Positive (FP) instances are those that the system under consideration identified incorrectly as abnormal mass but were actually normal occurrences, True Positive (TP) cases are those in which the current proposal correctly identified them as existing masses (BC), True Negative (TN) cases are those in which the proposed system incorrectly identified them as abnormal masses but which are actually normal occurrences.

### Ethics approval and consent to participate

The participants gave written informed consent before the collection of specimens. The protocol was approved by the Faculty of Medicine at Ain Shams University in Egypt and complied with the Declaration of Helsinki's Ethical Principles for Medical Research Involving Human Subjects. (No. P.T.REC/009/003156).

## Results

**Figure 4:**
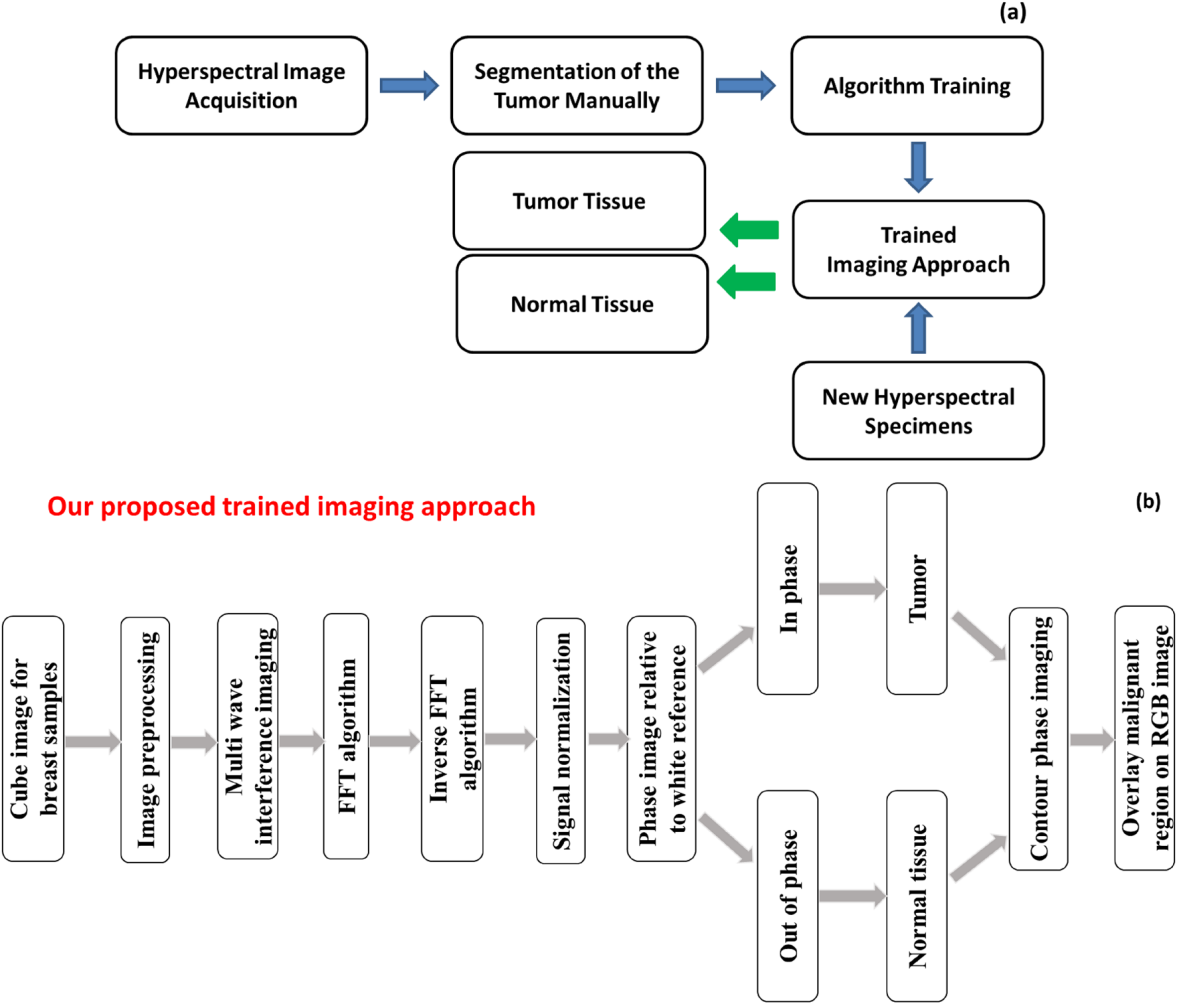
(**a**) The hyperspectral images tissue categorization technique methodology; (**b**) Flowchart illustrating the proposed trained imaging approach.

**Figure 5:**
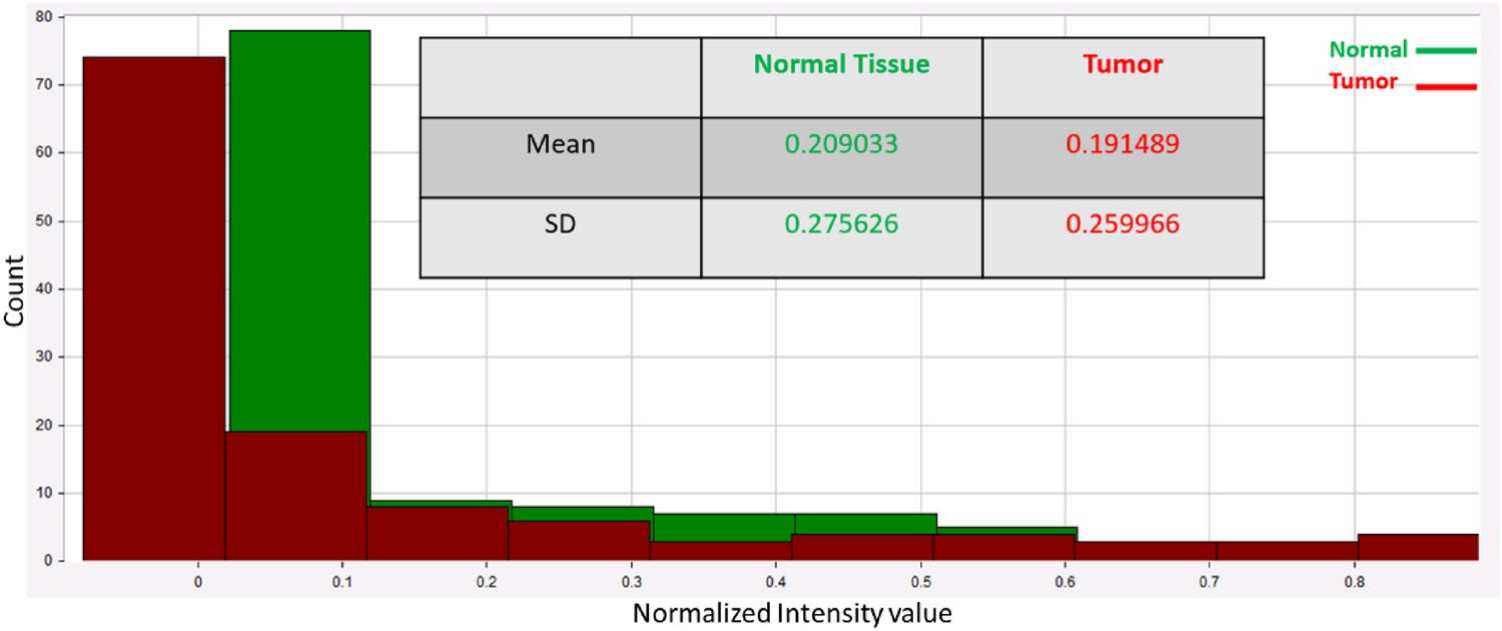
Average computed SD, Mean, and Histogram of the observed data for the examined breast tissue samples.

These studies are intended to show that the HS method can detect and categorize the tumor tissue based on their MWI reflectance spectra and dissociate from healthy tissues. The specimens were illuminated by a source of white polychromatic bulb (380–980 nm). After imaging setup calibration^30^, the ex-vivo HS imaging investigation included a total of 30 breast samples. We divide the tumor into a single group of HS pictures to train our classifier by analyzing already stained samples. A new round of HS images is processed using the learned algorithm. Automated tumor classification using the latest HS images. The trained algorithm's output is the outcome of the classification of both malignant and healthy tissue in the image. The methodology of the HS images tissue categorization approach is shown in Fig. 4a. The proposed trained imaging approach flowchart is depicted in Fig. 4b. Figure 5 illustrates our imaging approach capability to get descriptive statistical measures represented in Histogram, mean and standard deviation (SD) for the tested samples.

**Figure 6:**
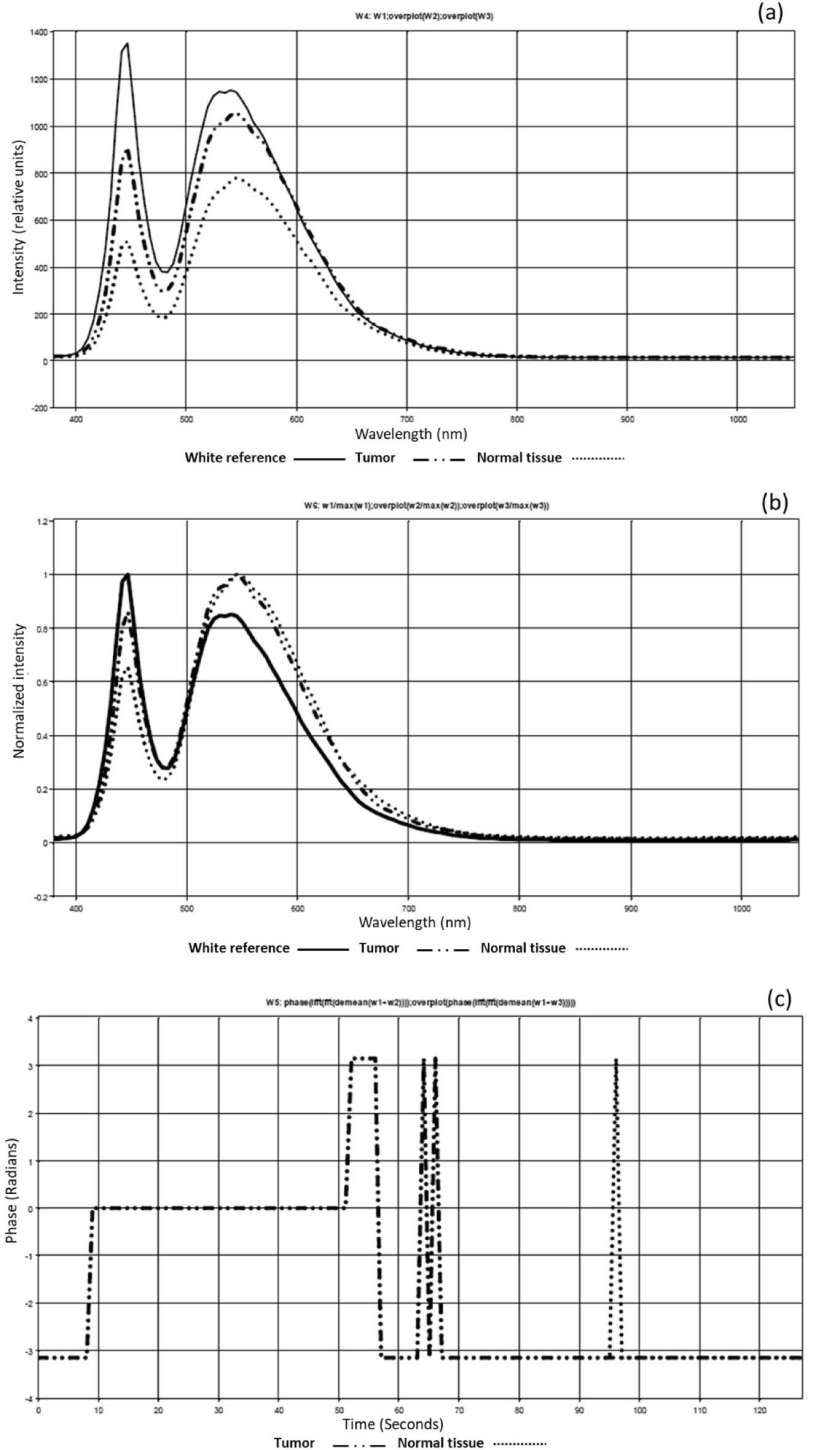
(**a**) The average diffuse reflected signals from white paper reference, tumor region, and normal region; (**b**) Phase shift analysis after signal normalization; (**c**) Absolute phase shift change measurement between normal and malignant tissue.

According to the proposed algorithm in Fig. 4b, our phase imaging processing method incorporates a critical step of signal normalization. This normalization is essential to eliminate system dependencies arising from amplitude fluctuations, ensuring that our analysis is focused primarily on phase information, which is crucial for our research. Furthermore, it is important to emphasize that our proposed clustering approach for grouping malignant breast tissue in the context of BC detection relies heavily on quantitative phase measurements. These measurements are derived from the MWI patterns obtained from the cube image relative to the white reference. By quantifying phase differences, we are able to accurately differentiate between malignant and normal tissue, and this forms the core of our innovative approach for BC assessment. In our trained algorithm, we employed a dataset consisting of 30 samples, with all samples confirmed to contain malignant regions through pathology examination. Specifically, each of these samples was pathologically verified to have malignant tissue. While all the samples included malignancy, we placed emphasis on ten of them, which were stained by pathologists. The staining of these ten samples allowed us to provide a comprehensive assessment of our work, highlighting the success of our trained algorithm based on MWI and the cube images captured with the HS camera. Our classification algorithm was designed based on HS and the phase measurements obtained from the cube images using the HS camera. The classifier was trained on this subset of ten stained samples, and each sample was run three times to account for variability and ensure the robustness of our classifier. This approach helped the algorithm learn the characteristics of malignant and healthy tissue regions based on the MWI phase measurements. The training process allowed our algorithm to identify patterns and features indicative of BC in the images using our automatic image processing approach based on FF transform methodology. Once the classifier was trained, we employed it to evaluate the remaining unstained samples. The outcome of this evaluation was compared to the pathology findings. Our robust validation process, involving all 30 samples, including ten stained samples, and twenty unstained samples, clearly illustrated the effectiveness of our MWI-based classification approach for BC detection. Employing our MWI approach for BC detection, which utilizes blue and red images in relation to a white paper reference, we were able to analyze interference patterns using the FF transform method. This method provided us with depth-resolved information about the tissue, enabling the effective detection and characterization of breast tumors. Figure 6a–c illustrate the average diffuse reflected signals from the white paper reference, the tumor region, and the normal region. It highlights variations in the signals, presents the signals from the three regions after signal normalization, clearly revealing the phase shift differences between them. Additionally, it showcases the absolute phase shift change measurement between normal and malignant tissue, assessing the variance in phase measurements relative to the white reference, respectively.

**Figure 7:**
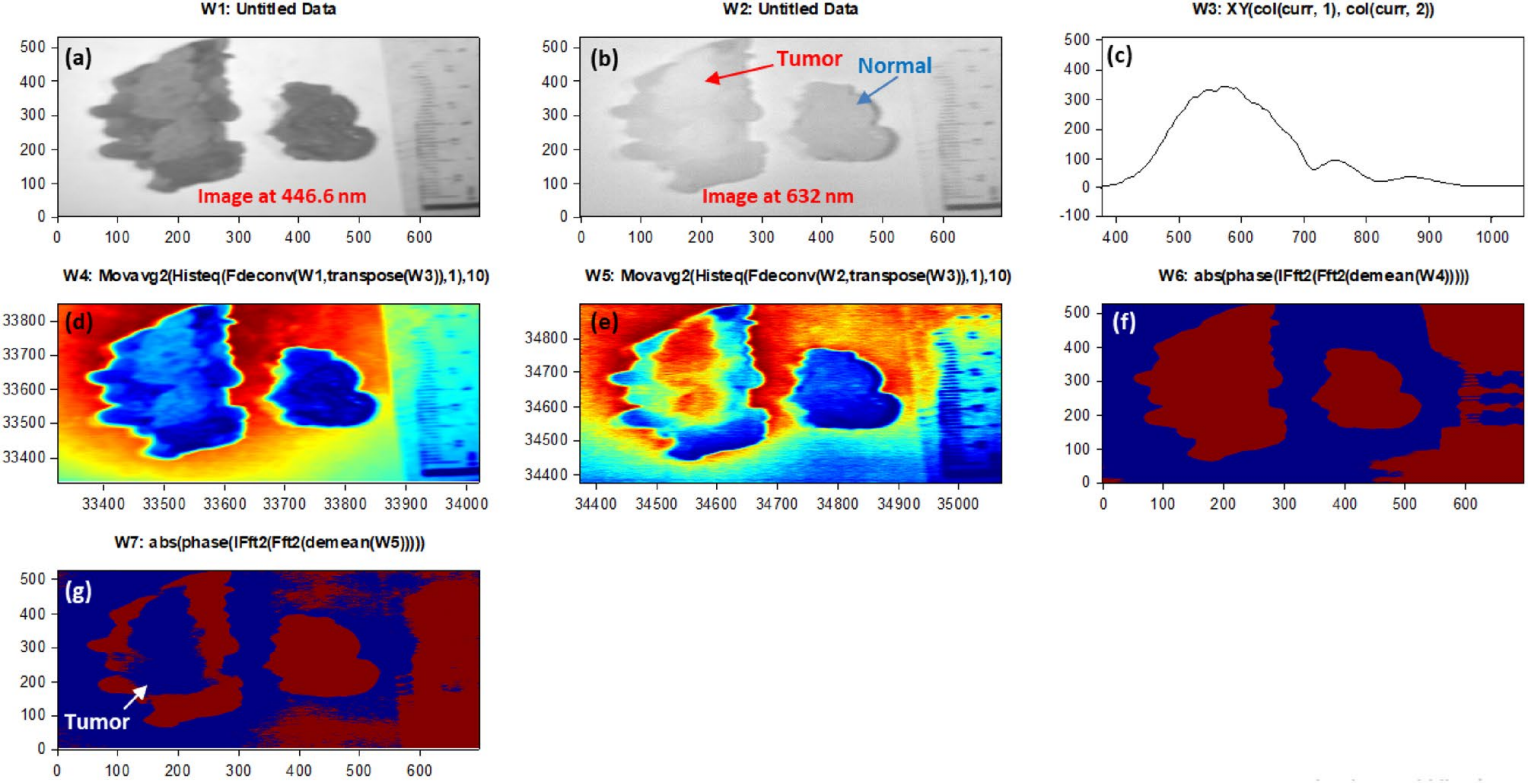
Findings of using the quantitative phase approach with image-preparation methods on stained sample #5 (**a**) The picture acquired at 446.6 nm by the HS imager;(**b**) The picture acquired at 632 nm by the HS imager;(**c**) The polychromatic light source's white reference response;(**d**) The 446.6 nm image after implementing preprocessing methodology ;(**e**) The 632 nm image after implementing preprocessing methodology,(**f**) Absolute phase shift calculations at the 446.6 nm image; (**g**) Absolute phase shift calculations at the 632 nm image (blue spot).

**Figure 8:**
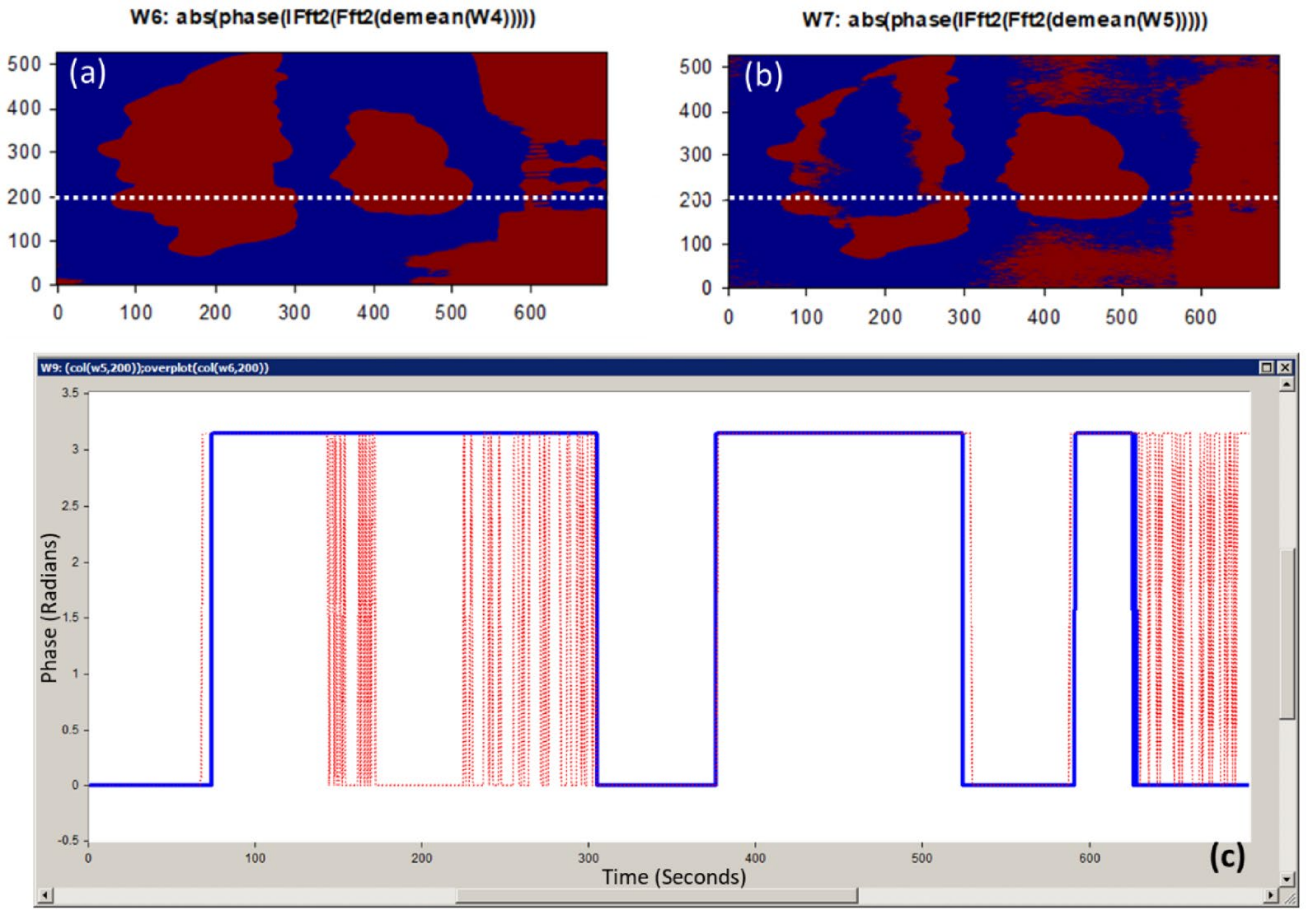
(**a**) The 446.6 nm image phase calculation at horizontal line = 200; (**b**) The 632 nm image phase calculation at horizontal line = 200; (**c**) The absolute phase-shift change between the 446.6 nm spectral image (blue line) and the 632 nm spectral image (red line) for sample #5.

According to Fig. 6, we could emphasize how signal normalization enhances the visibility of phase shifts between the signals. The phase shift between these signals becomes more apparent, providing essential data for subsequent analysis. Moreover, we confirmed the absolute phase shift change measurements between normal and malignant tissue. The variance in phase measurements relative to the white reference is assessed, highlighting the differences between these tissue types. This data is crucial for our proposed phase-based clustering approach for BC detection. Figure 7 shows the outcomes of utilizing our phase change strategy and image preparation method to the stained sample #5 for algorithm training purposes using the blue and red images at 446.6 nm and 632 nm, respectively. The output measured phase shift information is plotted to show the variance between the malignant tissue and the normal tissue relative to the white reference effect using the resulted processed images at the horizontal line # 200 for the two as shown in Fig. 8.

**Figure 9:**
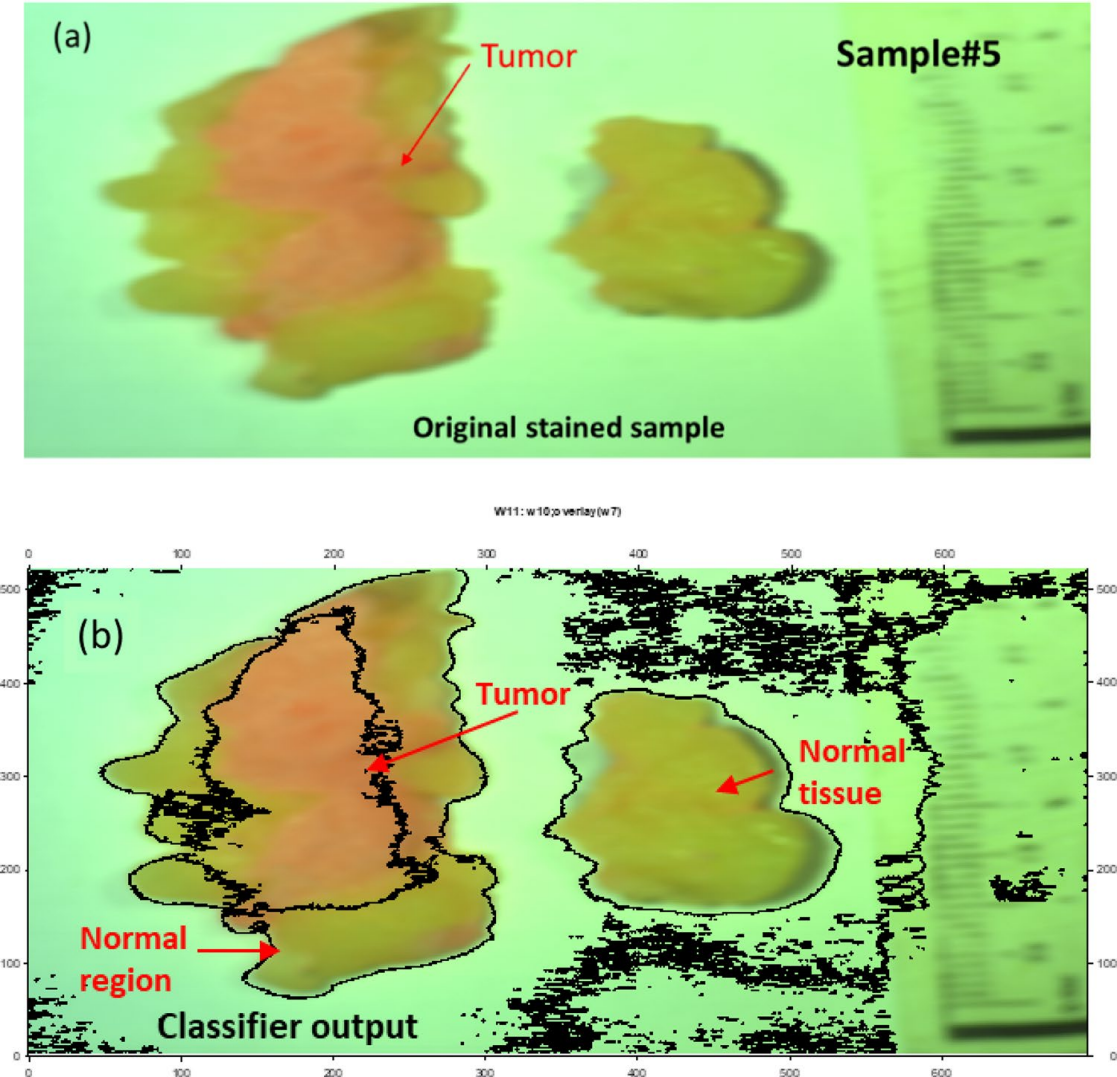
The HS image classification with automatic cancer tissue diagnosis on pathology specimen# 5. (**a**) The cancer is visible in the original stained sample. (**b**) Automatic phase computations found the majority of the malignant tissue.

Using the FF transform model and the inverse (Inv) FF transform, Fig. 7 shows how to apply our phase shift technique to the spectra pictures (446.6 nm and 632 nm). Figure 7c shows the calculated white reference effect that was used with deconvolution algorithm. As illustrated in Fig. 7d and e, we employed our unique preprocessing approach, which combines histogram equalization and MA filtering, to highlight and define the tumor for the two chosen spectral images. Figure 7f demonstrates how the deep tumor is not distinguished from the normal tissue regions using the 0.4466 µm spectral image. Figure 7g depicts the tumor determination from the normal regions using a 0.632 µm spectral image. According to the difference in penetration depth between the blue and red pictures and the white reference, shown in Fig. 8c, we could certainly define the phase shift data. One instance of the original stained breast and the categorization outcome are shown in Fig. 9. The pathology-stained sample's HS pictures were categorized using our approach. A pathologist stained the cancerous tissue. The tumor location was clearly discernible using the HS classification method. High *Spec* and *Sen* are required for the test, with the response assessed at a wavelength that goes into the tissue depths. Diagnosing breast cancer is a delicate procedure that has to be handled cautiously. After measuring the absolute phase shift on the original stained sample, we were approximately able to overlay the 632 nm picture successfully.

According to our trained algorithm, explained in Fig. 4, and the proposed phase MWI imaging approach, we were successful in detecting the tumor with a minor FN region and nearly small FP area (Sample # 5). The procedure of diagnosing BC is delicate and must be handled with care, hence the test's *Sen* and *Spec* levels should be high using the binary classification approach^44–47^. By computing the TP, TN, FP, and FN values for the evaluation at 632 nm, pixel accuracy is assessed. If a pixel was a malignant pixel in the histology examinations constructed map but was not recognized as such, the detection was regarded as a FN. If a pixel was mistakenly identified as cancerous tissue when it was discovered, the discovery was labelled as a FP. These steps are carried out for each of the ten stained samples separately. We conducted data acquisition three times for each of the ten stained samples to ensure the robustness and repeatability of our results. This multiple acquisition strategy allowed us to assess the consistency of our findings and the reliability of our trained algorithm across different runs. Our classifier achieves for this sample FN ratio, FP ratio, *Sen* and *Spec* of 8.4%, 4.8%, 91.6% and 95.2%, respectively. The *Sen* and *Spec* average calculation were 90.9% and 94%, respectively for the ten stained samples as displayed in Table 1.

**Table 1:** Average *Sen* and *Spec* of MWI phase categorization for ten stained samples at 632 nm (based on three runs per sample).

Specimen	1	2	3	4	5	6	7	8	9	10	Mean
*Sen (%)*	98.93	91.5	88.3	91.5	91.6	88.7	87	90.5	90.8	90.2	90.903
*Spec (%)*	97.3	93.5	93.5	93	95.2	92.8	94.1	94.1	93.6	93	94.01
FN ratio* (%)*	1.07	8.5	11.7	8.5	8.4	11.3	13	9.5	9.2	9.8	9.097
FP ratio* (%)*	2.7	6.5	6.5	7	4.8	7.2	5.9	5.9	6.4	7	5.99

**Figure 10:**
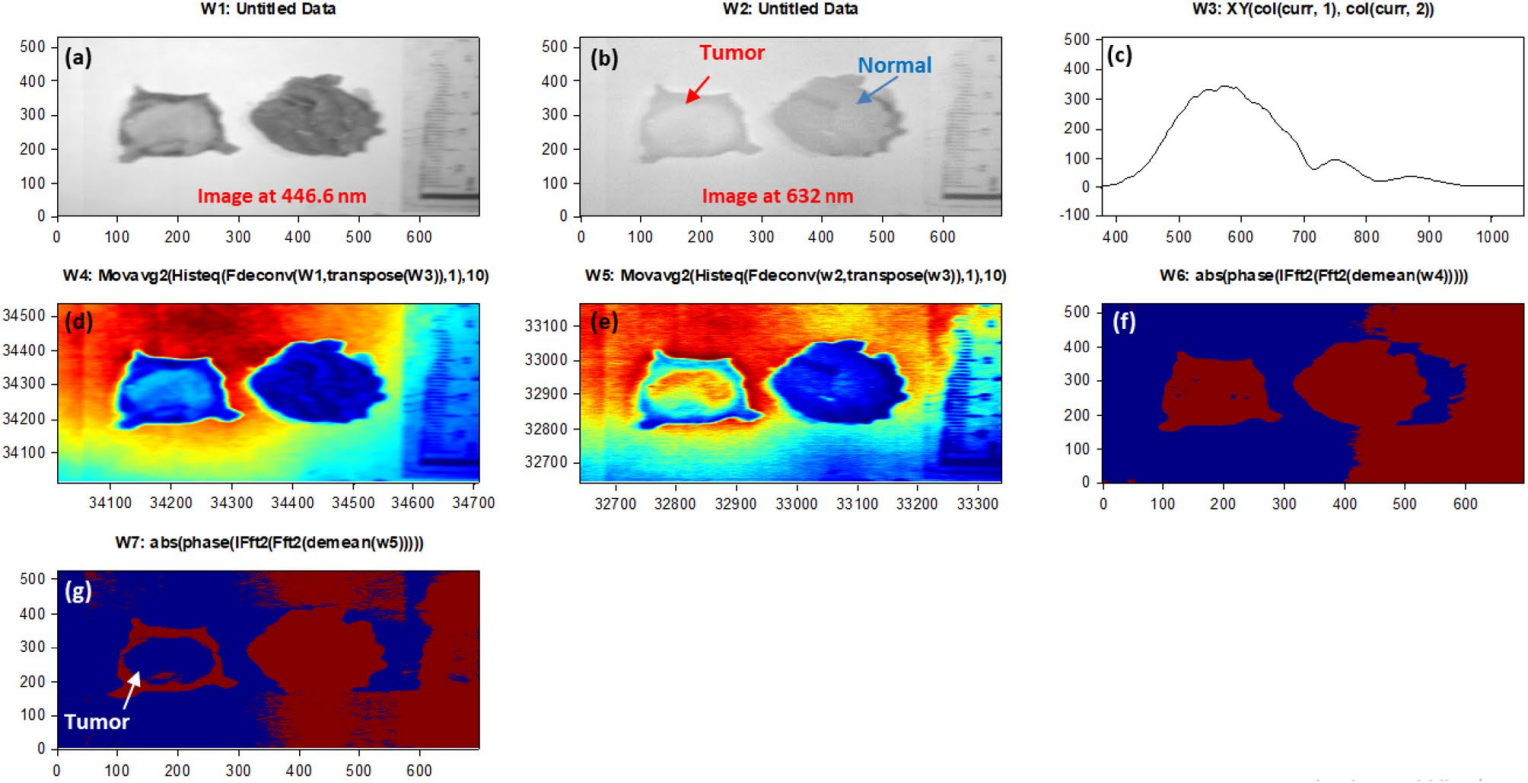
Outcomes of applying image-preprocessing methodology and quantitative phase method on unstained sample #6 (**a**) The picture acquired at 446.6 nm by the HS imager; (**b**) The picture acquired at 632 nm by the HS imager; (**c**) The polychromatic light source's white reference response; (**d**) The 446.6 nm image after implementing preprocessing methodology ;(**e**) The 632 nm image after implementing preprocessing methodology, (**f**) The 446.6 nm image phase calculation; (**g**) The 632 nm image phase calculation (blue spot).

We could apply our methodology to unstained, unknown breast samples, including breast sample #6, using our classifier. The results of applying our proposed technique using quantitative phase approach to the unstained, unknown breast biopsy that belongs to sample #6 are illustrated in Fig. 10.

**Figure 11:**
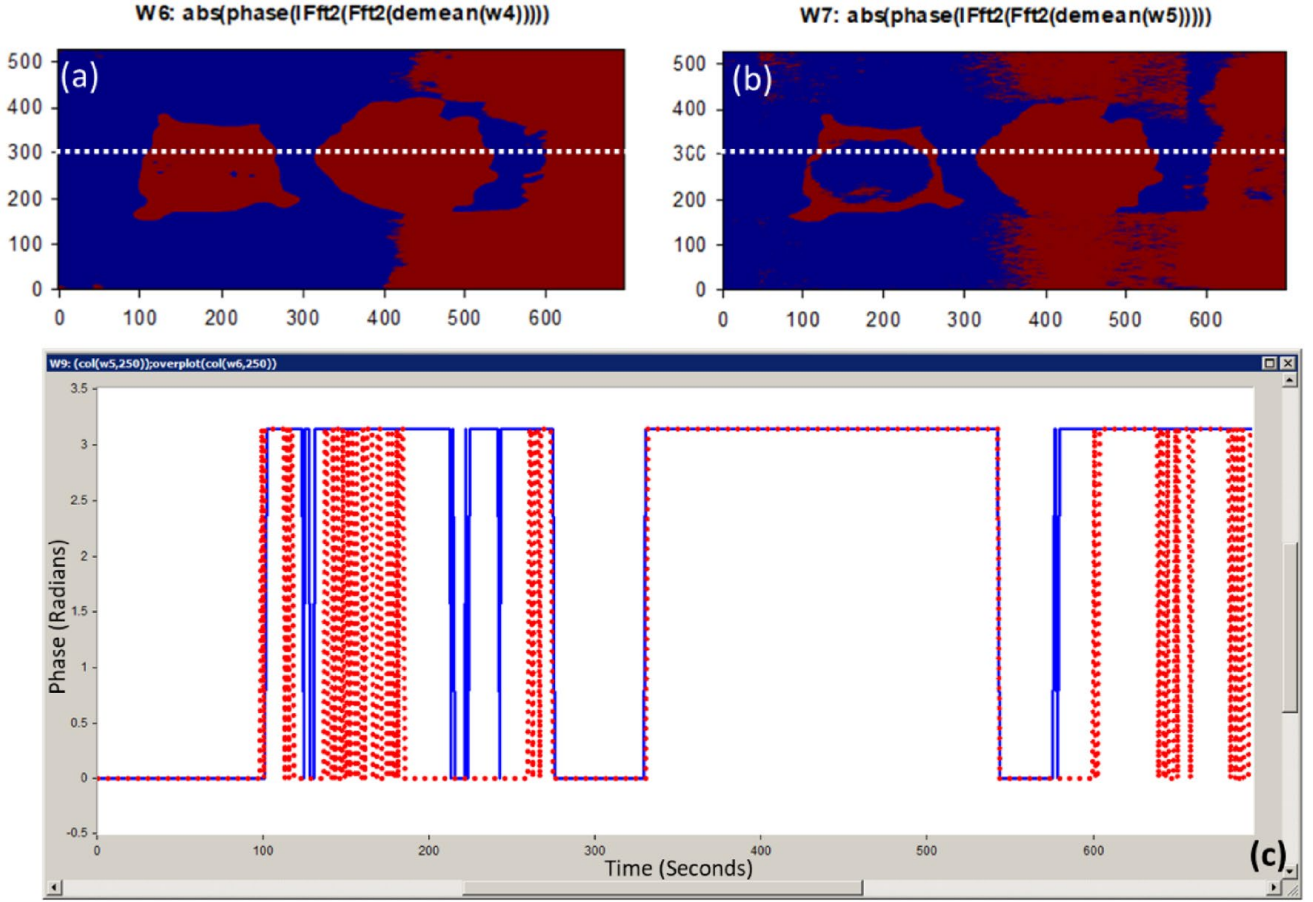
(**a**) The 446.6 nm image phase calculation at horizontal line = 300; (**b**) The 632 nm image phase calculation at horizontal line = 300; (**c**) The absolute phase-shift variance for unknown breast sample #6 between the 446.6 nm and 632 nm spectral images (blue and red lines, respectively).

**Figure 12:**
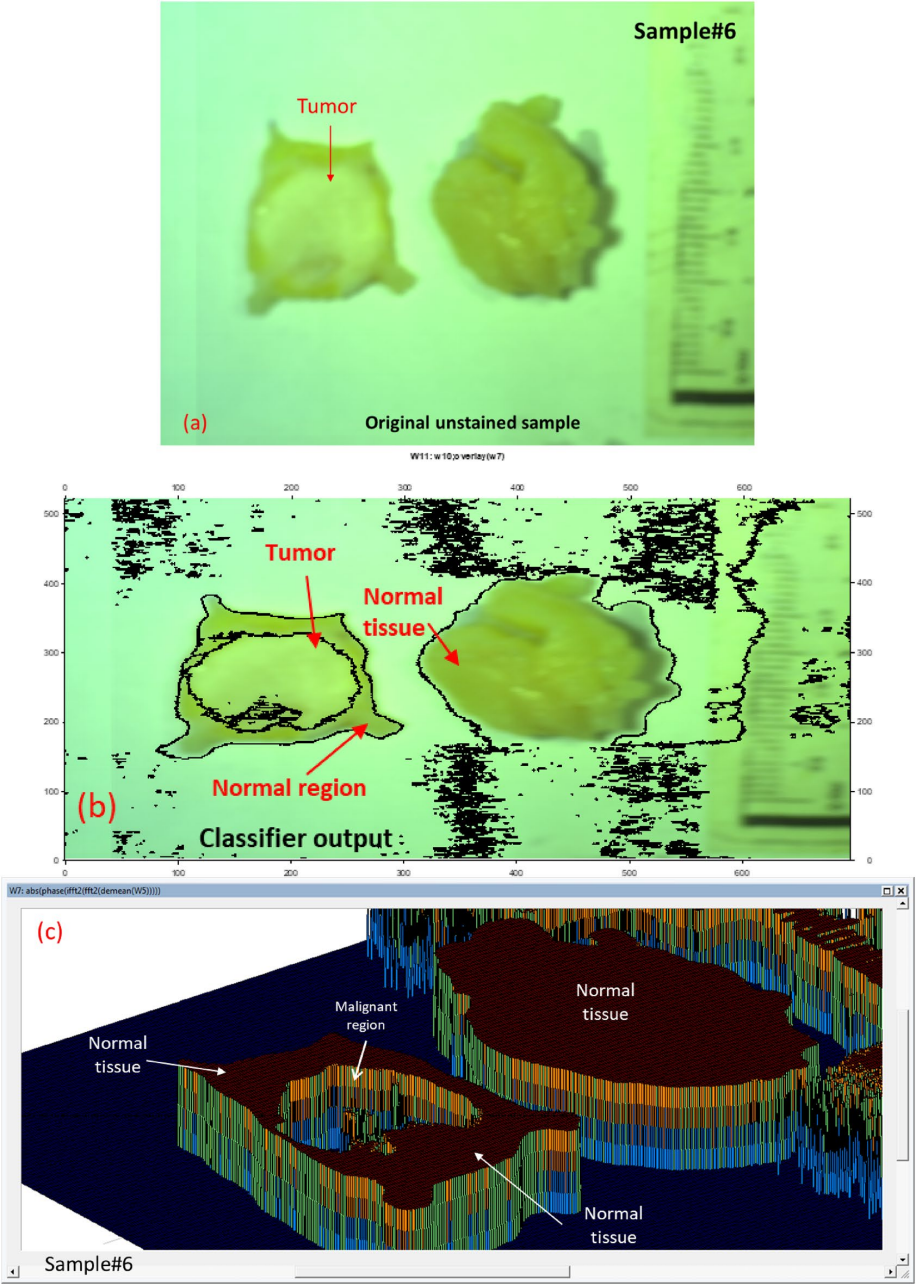
HS image classification for automatic cancer tissue diagnosis on pathology specimen#6. (**a**) The cancer is unknown in the original breast sample; (**b**) The detection of the tumor tissue based on our automatic MWI phase technique; (**c**) The 3D phase imaging findings for this specimen showing the layers of malignancy.

Confirming our imaging approach on the suitable HS spectral response (446.6 nm and 632 nm) for tumor detection, we applied our MWI phase approach on sample#6. Figure 10 (a) and (c) show the selective 446.6 nm spectral image and the improved image quality output using our imaging algorithm. The 632 nm spectral image for this case study and then applying image preprocessing are clearly described in Fig. 10b and d, respectively. Figure 10f and g show the use of our MWI phase imaging technique to achieve tumor and normal tissue grouping based on the two selective spectral images (446.6 nm and 632 nm). In Fig. 11c, the variation between the healthy tissue and the malignant tissue in relation to the white reference effect is displayed using the output measured phase shift information from Fig. 11a and b at the horizontal line # 300. We overlaid the 632 nm image on the unknown breast sample image after detecting the absolute phase shift between normal and malignancy tissue as depicted in Fig. 12. Figure 12c shows the outcomes of applying our proposed 3D phase imaging method to sample #6, clustering the malignant regions.

Figure 11 shows that because of the difference in absorption coefficient in both the blue and red signals, we could in fact define the phase shift data at different layers from tissue. According to Fig. 12, our classifier could automatically identify and cluster the tumor location in the breast samples using the MWI phase shift information and build a 3D phase imaging for the tissue clustering the BC regions from the normal tissue. Examiners' preliminary judgments may be improved by our procedures.

## Discussion

In this study, we developed and evaluated the MWI approach for BC detection by utilizing cube images obtained from HS imaging. Our approach involves the implementation of an automatic image processing method rooted in quantitative phase imaging. Our phase analysis approach, when combined with developing imaging modalities, provides a wide variety of potential medicinal applications. This method might help physicians provide preliminary estimations for cancer patients. Early cancer detection and therapy increase the likelihood that a patient will survive and the possibility of a full recovery. In addition to the low expense and great responsiveness associated with the lack of staining, the prospect of a highly automated method may have a significant influence on pathology on a worldwide market. Cancerous tissue is identified pixel by pixel using the suggested MWI phase classification technique. Because the identification of one diseased pixel is independent of surrounding pixels, it has the potential to identify invasive malignant tissue of various sizes and forms. A surgical biopsy needs to be collected and transported to a pathology lab for processing and evaluation by a pathologist. MWI phase imaging may be able to save significant time throughout surgery; that could clearly be a huge gain given that the entire process can take hours. As compared to typical interferometric imaging setups, this imaging approach has several advantages. Most importantly, the necessity for expensive and accurate optical components is eliminated. There is also a significant amount of computing capability to separate the amplitude and phase information in the produced cube pictures. So, in order to engage with the specimens, we constructed a practical HS framework where the SOC710 HS system has been lit using a white polychromatic lamp source. We used our optical diagnosis method to examine thirty breast samples, detecting the optimum surface and deep wavelengths to apply our MWI approach. Our initial findings found that red light at 632 nm is able to penetrate deeper into biological tissue due to its lower scattering coefficients. This makes it more effective for imaging deeper tumors in breast tissue. In the meantime, we observed that blue light at 446.6 nm is more easily absorbed by biological tissue and can be useful for imaging superficial structures or detecting changes in tissue morphology or cellular structure. By analyzing the interference pattern between these two wavelengths relative to the white reference effect, we were able to calculate the phase difference between them. This phase difference was then used to calculate the tissue refractive index, which can provide information about its composition and structure.

**Figure 13:**
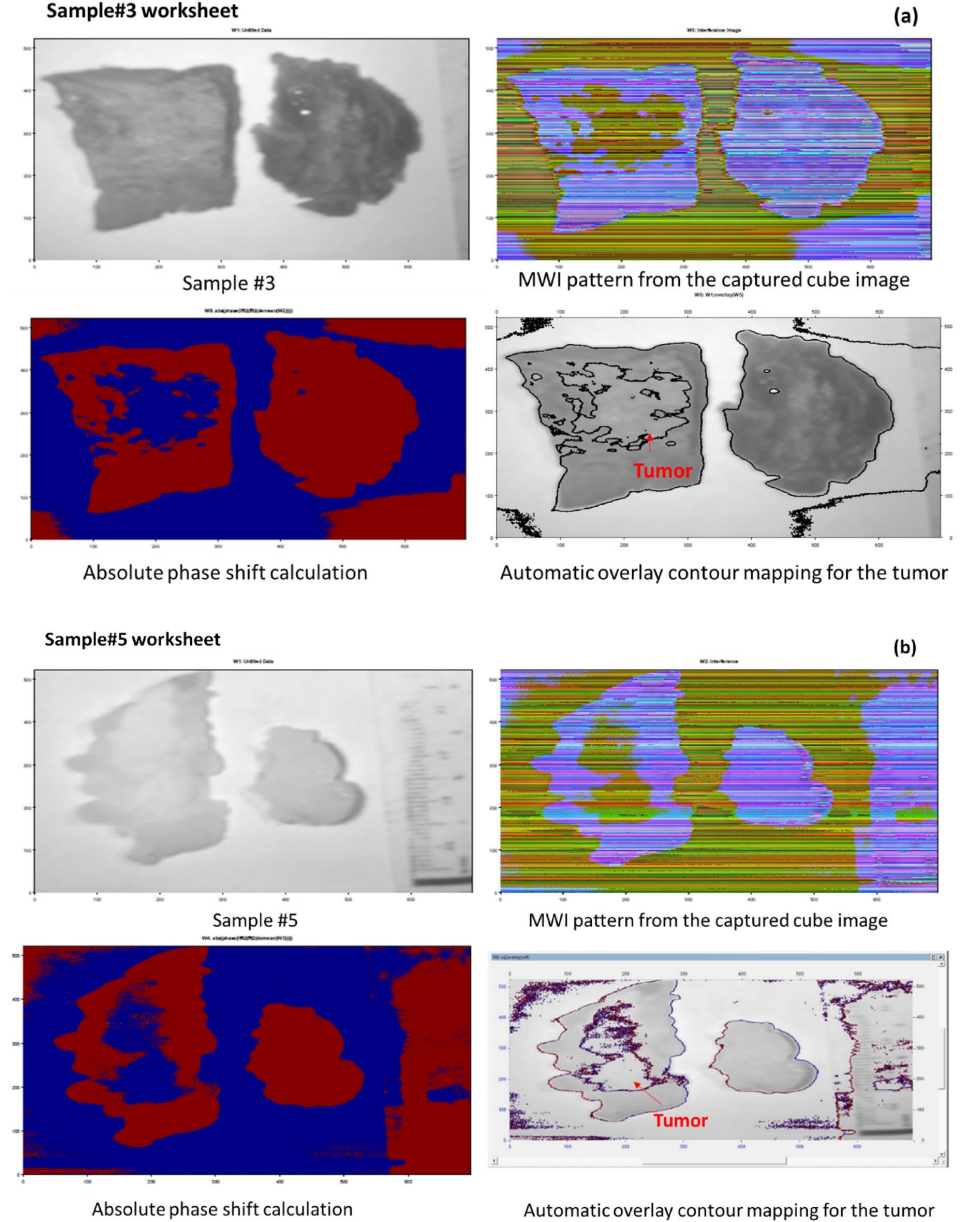

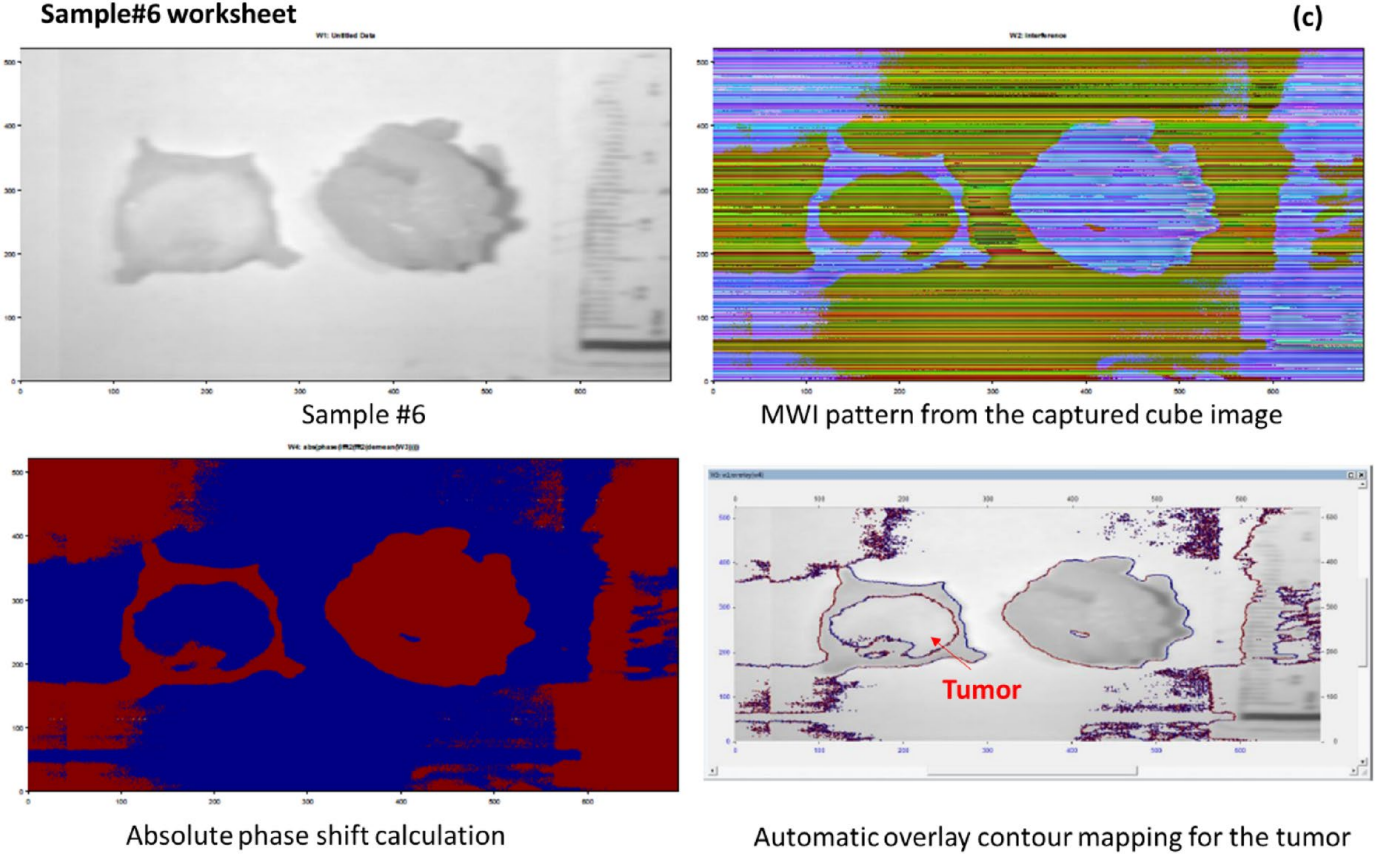
(**a**–**c**) Illustration of the MWI-based tumor location phase classification approach for grouping malignant tissue using data extracted from cube images of stained samples 3 and 5, and unstained sample 6, respectively.

Since the BC index of refraction is higher than the normal tissue refractive index^48–51^, and because the scattering characteristics of tissue cause light to disperse throughout the tissue, which ultimately results in a decrease in energy density as depth is increased^52,53^, So, we built our algorithm to be able to identify cellular and subcellular components within specimens based on the arrangement of refractive index^54,55^. Breast biopsies' refractive index maps pinpoint the areas of abnormal cells, which are helpful for BC evaluation and future prediction. The malignant areas in breast biopsies correspond with the spatial changes in refractive index recorded by our proposed MWI phase imaging approach. The advantage of using MWI is that it allows for a more accurate and detailed analysis of tissue properties compared to single-wavelength techniques. By using multiple wavelengths (446.6 nm and 632 nm), we can obtain information about both the tissue's scattering and absorption characteristics. Additionally, the use of interference patterns allows for precise measurements of phase differences, which can reveal details regarding the composition and structure of tissues. Finally, by using FF transform method to analyze the interference pattern, it may get a high-resolution spectral picture of the tissue sample, allowing for detailed analysis of its optical properties. Figure 13a–c depict our promising proposed MWI phase approach for categorizing tumor locations based on MWI data extracted from the acquired cube images of stained samples 3 and 5, and unstained sample 6, respectively.

Before converting the resulting spectrum image to the frequency domain, it must first be improved. Histogram equalization and MA filtering techniques were all used in our imaging strategy. The FF transform method is the foundation of our strategy for switching to the frequency domain. Using our Inv FF transform technique, we were able to eventually obtain our original processed photos in the temporal domain. This technique was mostly based on the DADiSP 6.5 software. Our method for processing images helped to provide highly accurate phase measurements with sub-wavelength resolution that could be utilized to tell the tumor from the surrounding healthy tissue.

Our training system successfully reached high classification accuracy. Prior to running our algorithm, we applied it to ten stained samples. The HS classification technique enabled precise localization of the tumor. For *Sen* and *Spec*, the average calculation was 90.9% and 94%, respectively. On one tested stained sample, the *Sen*, *Spec*, FN ratio, and FP ratio of our classification model were 8.4%, 4.8%, 91.6%, and 95.2%, respectively. We were able to correctly overlay the 632 nm picture over the original stained sample photo (stained sample #5) after detecting the absolute phase shift data. The MWI phase approach classifier was able to automatically detect and categorize the tumor location in the breast samples at depths with unstained, unidentified breast tissues. This automatic proposed methodology is trustworthy and may be utilized as a tool to help a surgeon during surgery decide and examine the resection edges in real time. Our MWI phase imaging technique, along with a captured image that only operates at a specific wavelength, might aid examiners in making first assessments.

## Conclusion

Multi-Wavelength Interference phase imaging approach is a promising method for detecting BC that may lead to earlier identification and better treatment outcomes. The technique has been demonstrated to be effective in detecting BC both in vitro and in vivo, and it has the advantage of being non-invasive. A precise phasing imaging-based technique based on the FF transform was used to classify cancer tissue. The methods for quantitative phase identification and MWI may be utilized to automatically identify BC, according to our methodological approach. The proposed technique using MWI phase imaging based on diffuse reflection HS imaging has the potential to revolutionize BC screening and diagnosis and would enable surgeons to examine and evaluate a substantial portion of tissue without the need to remove any biopsy for pathological study. Moreover, our proposed FF transform approach provided the unique privilege of obtaining 3D plotting for the investigated tissue and making a trace for the malignant location. Enhancing a surgeon's visual talents might also be a major benefit. One of the benefits associated with this approach is how easily phase data can be used to confirm the spectrum changes of different tissue types and depths. As our method permits automatic continuous inspection of cancer tissue that is suspected of being malignant without interfering with surgery at various depths, it may be used as a feasible biopsy technique. By combining our effective imaging technique during surgery with a special conventional RGB camera that only operates at specific wavelengths, a new and efficient method for application in early tumor diagnosis may be created.

## Data Availability

The authors stated and declare that all the datasets used and/or analyzed during the current study are available from the corresponding author on reasonable request to preserve the copyright. The authors stated and declare that all code exists and is available.
